# Integrative assessment of congestion in heart failure using ultrasound imaging

**DOI:** 10.1007/s11739-024-03755-9

**Published:** 2024-09-05

**Authors:** Valerio Di Fiore, Lavinia Del Punta, Nicolò De Biase, Pierpaolo Pellicori, Luna Gargani, Frank Lloyd Dini, Silvia Armenia, Myriam Li Vigni, Davide Maremmani, Stefano Masi, Stefano Taddei, Nicola Riccardo Pugliese

**Affiliations:** 1 Department of Clinical and Experimental Medicine, University of Pisa, Via Roma 67, 56124 Pisa, Italy; 2 School of Cardiovascular and Metabolic Health, University of Glasgow, Glasgow, G12 8QQ UK; 3 Thoracic and Vascular Department, Azienda Ospedaliero-Universitaria Pisana, Via Paradisa 2, 56124 Pisa, Italy; 4 Istituto Auxologico IRCCS, Centro Medico Sant’Agostino, Via Temperanza, 6, 20127 Milan, Italy

**Keywords:** Heart failure, Ultrasound, Congestion

## Abstract

In heart failure (HF), congestion is a key pathophysiologic hallmark and a major contributor to morbidity and mortality. However, the presence of congestion is often overlooked in both acute and chronic settings, particularly when it is not clinically evident, which can have important clinical consequences. Ultrasound (US) is a widely available, non-invasive, sensitive tool that might enable clinicians to detect and quantify the presence of (subclinical) congestion in different organs and tissues and guide therapeutic strategies. In particular, left ventricular filling pressures and pulmonary pressures can be estimated using transthoracic echocardiography; extravascular lung water accumulation can be evaluated by lung US; finally, systemic venous congestion can be assessed at the level of the inferior vena cava or internal jugular vein. The Doppler evaluation of renal, hepatic and portal venous flow can provide additional valuable information. This review aims to describe US techniques allowing multi-organ evaluation of congestion, underlining their role in detecting, monitoring, and treating volume overload more objectively.

## Introduction

Heart failure (HF) is a complex clinical syndrome characterised by the presence of symptoms (e.g. dyspnea, fatigue) and signs (e.g. elevated jugular venous pressure, pulmonary crackles, and peripheral oedema) due to structural and/or functional abnormalities of the heart that lead to inadequate cardiac output and/or abnormally elevated filling pressures [1]. Congestion, defined as extracellular fluid accumulation, is a central feature of HF and carries serious prognostic implications [1, 2]. From a pathophysiologic point of view, congestion develops due to salt and water retention by the kidney, resulting in elevated ventricular filling pressures; this, in turn, leads to augmented pressures in the pulmonary and/or systemic venous compartment (i.e., intravascular congestion) and subsequent fluid leakage in the extravascular space (i.e., extravascular or tissue congestion) [3]. There is little evidence to suggest that treatment targeting congestion (e.g., with diuretics) modifies the natural history of HF [1]. Still, decongestive therapies are a cornerstone in the treatment of symptoms and signs in both acute and chronic HF, and are lifesaving for patients in pulmonary oedema [1]. Furthermore, timely detection and management of elevated venous pressures and fluid accumulation have been shown to offset the risk of HF-related events [4–6]. In the clinical setting, the physical examination remains an essential first step in assessing congestion in patients with suspected or established HF [6]. However, clinical assessment alone has inherent limitations [3], highlighting the importance of a more sensitive, objective evaluation of congestion [7, 8]. Cardiomyocytes release natriuretic peptides in response to pressure and volume overload; their measurement is recommended in the diagnostic workup of suspected HF and offers valuable prognostic information [9]. However, natriuretic peptide levels are influenced by various factors, such as age, body mass index, and atrial fibrillation [1], that may hamper the reliability of this bio-humoural marker as a proxy for fluid overload, and laboratory testing is time-consuming [10, 11]. The gold standard to assess congestion is right heart catheterisation (RHC) [12], which enables the direct measurement of right atrial pressure (RAP), commonly used as an index of central venous pressure (CVP), and pulmonary capillary wedge pressure (PCWP). However, routine RHC has several limitations due to its invasive nature. Ultrasound (US) offers a non-invasive, real-time evaluation which can assist clinicians in diagnosing congestion [10], monitoring the effect of diuretic therapy [10], and predicting outcomes [3] at the same time as an echocardiogram. Integrating these methods addresses the limitations of relying on a single diagnostic tool, offering a more accurate and comprehensive assessment of a patient’s hemodynamic status [3, 13].

**Fig. 1:**
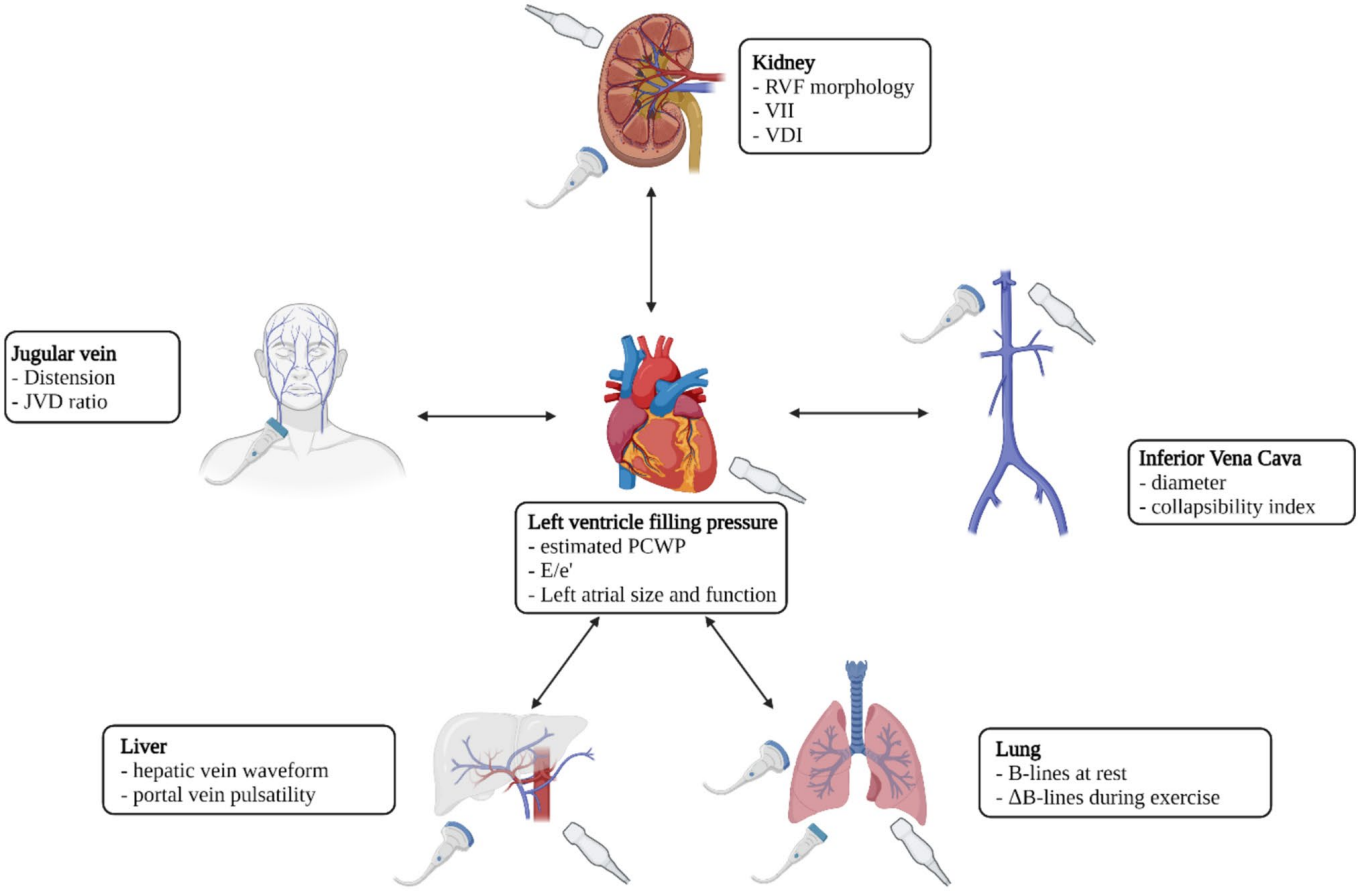
Ultrasound techniques to assess congestion in HF. *HF* heart failure, *JVD* jugular vein distension, *PCWP* pulmonary capillary wedge pressure, *RVF* renal venous flow, *VDI* venous discontinuity index, *VII* venous impedance index

The US-based assessment of congestion aims to estimate ventricular filling pressures [10] and identify lung parenchymal [14] and systemic venous congestion through the evaluation of the inferior vena cava [15], internal jugular veins [10], portal and hepatic veins [16], and intrarenal venous flow [10] (Fig. 1). With this background and drawing upon recent advancements in US technology and clinical guidelines, this review aims to summarize the role of novel US techniques in the early detection, monitoring, and management of fluid overload.

## Ultrasound methods to detect and quantify congestion

*Cardiac US.* Transthoracic echocardiography enables a detailed assessment of cardiac structure and function, and a non-invasive estimation of both left ventricular filling pressures (LVFP) and pulmonary pressures [12]. The ratio of early transmitral flow velocity to early diastolic myocardial velocity (E/e’) is generally accepted as a proxy for LVFP and used to evaluate diastolic dysfunction [17, 18]. Assessment of left atrial size and function are also important markers that estimate LVFP and stratify cardiovascular risk [19, 20]. However, quantification of LVFP using echocardiography lacks universal agreement [10]. More recently, an equation has been proposed to estimate pulmonary capillary wedge pressure from standard echocardiographic variables [12]. Said equation was validated against the gold standard of RHC, which shows the potential utility of echocardiography in this setting, especially when an invasive evaluation is not necessary (e.g., in low-risk patients with stable HF), is unfeasible or carries excessive risk. On the other hand, measuring the peak tricuspid regurgitation velocity allows the estimation of the systolic pulmonary arterial pressures. Elevated pressures in the pulmonary circulation are associated with fluid extravasation in the lung interstitium and parenchyma [21].

**Fig. 2:**
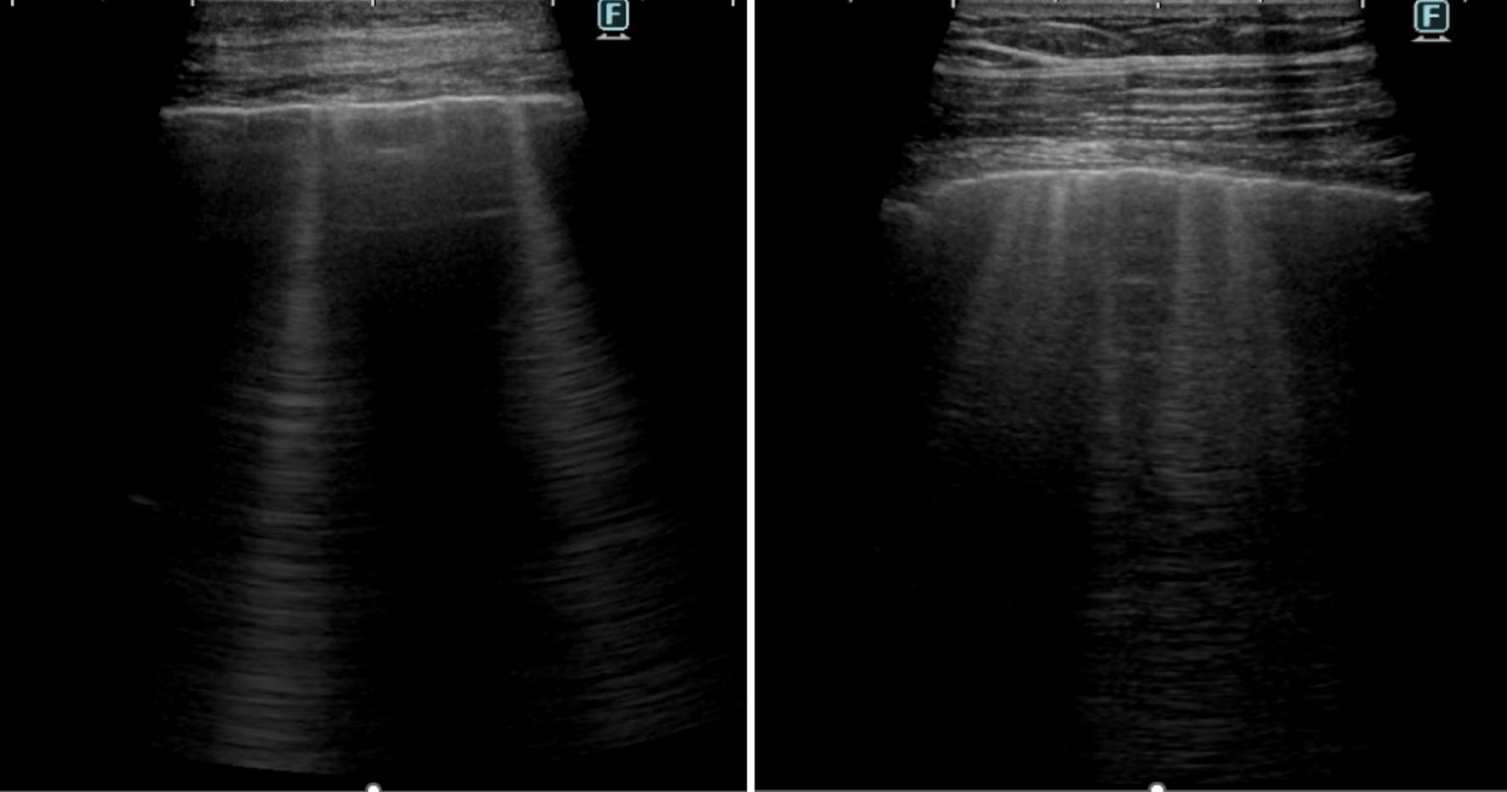
Lung ultrasound (LUS). Left. LUS evaluation of a single zone where 2 B-lines can be seen. Right. LUS evaluation of a single zone where at least 5 B-lines can be seen (i.e., at least 50% of the sector is covered by B-lines), suggesting pulmonary congestion

*Lung ultrasound (LUS).* LUS is a valuable tool for detecting and quantifying extravascular lung fluid by evaluating B-lines. B-lines are vertical hyperechoic reverberation artefacts arising from the pleural line and extending to the bottom of the ultrasound screen and indicate the presence of extravascular lung water [14] (Fig. 2). In patients with HF, B-lines exhibit rapid changes in response to therapies that modify intravascular volume or pulmonary pressures. For instance, their number reduces during renal dialysis or after treatment with loop diuretics, while they might increase with exercise [21–27]. In patients presenting to the emergency room with acute dyspnoea, LUS has shown superior sensitivity and specificity in diagnosing interstitial pulmonary oedema compared to clinical examination or chest X-ray [28, 29]. However, B-lines are not exclusive of HF, as they can also appear in non-cardiogenic pulmonary oedema (such as acute respiratory distress syndrome) [30] and interstitial lung disease [30, 31], and might be low in patients who are obese [32].

From a technical point of view, LUS can be performed rapidly with the patient in the supine position, placing the phased array or linear transducer either perpendicularly or transversely to the ribs on the anterior thorax, preferably using an eight-zone protocol (four symmetrical zones per each hemithorax) [10]. Maintaining consistent patient positioning during serial exams is advisable, as the count of B-lines can vary with changes in position, typically increasing when the patient is supine [33]. There are several methods to evaluate B-lines, which include both scoring and counting approaches. In scoring methods, a specific intercostal space with a minimum number of B-lines (i.e., ≥ 3) is marked as positive, and the total number of positive zones is recorded [28, 34]. Alternatively, B-lines can be individually counted within a specific area of the chest or approximated by the proportion of the sector area they cover beneath the pleural line [35, 36]. The quantitative technique is more precise and reliable in terms of consistency within and between different observers [37, 38]. A cut-off value of ≥ 3 B-lines in at least two zones per hemithorax has been proposed to identify acute HF with high sensitivity and specificity [28]. In chronic HF, a higher number of B-lines is associated with an increased risk of subsequent HF admissions and mortality, even in patients with few or no symptoms [3, 23, 39].

Inferior vena cava (IVC). The diameter of the IVC and its respiratory changes are regarded as indicators of CVP [40]. From a pathophysiologic point of view, a high CVP leads to IVC distention and, eventually, to reduced collapsibility during inspiration [40]. Thus, US evaluation of the IVC is valuable for assessing systemic venous congestion, monitoring a patient’s response to diuretic treatment, and predicting outcomes [40]. Importantly, many ambulatory patients with chronic HF who are presumed to be clinically free from congestion are subsequently found to have a dilated and/or non-collapsing IVC, which is associated with elevated natriuretic peptides and an increased risk of poorer outcomes, regardless of LVEF [3, 39, 41]. Furthermore, in patients discharged after a hospitalisation due to acute decompensated HF, the presence of a persistently dilated IVC before discharge is associated with a high risk of rehospitalisation [42]. Surprisingly, invasive studies on patients undergoing RHC revealed only a modest correlation between RAP and the diameter of the IVC measured by echocardiography [40]; this correlation is even weaker in patients mechanically ventilated, as positive intrathoracic pressures generated by mechanical ventilation cause IVC dilation and reduce its collapsibility [43].

**Fig. 3:**
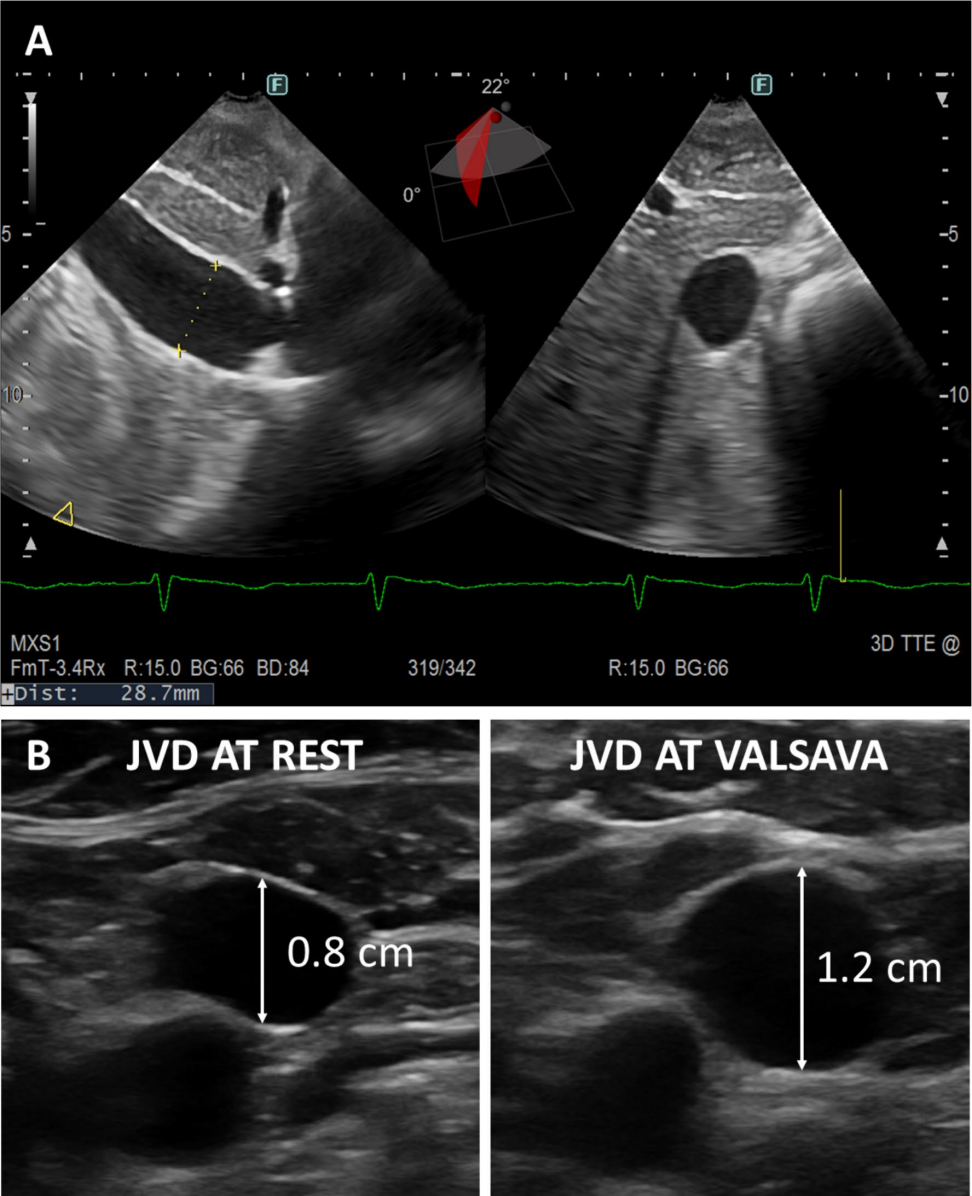
**A** Ultrasound assessment of the inferior vena cava.** B** Ultrasound assessment of the internal jugular vein at rest and after Valsalva (Jugular Venous Distensibility ratio ~ 1.5, which suggests severe congestion)

To perform an accurate ultrasound evaluation of the IVC, a phased array or curvilinear transducer should be used with the patient lying supine. The diameter of the IVC should be measured in the subcostal long-axis view, 1–2 cm proximal to its connection with the right atrium. The IVC diameter should be measured throughout the respiratory cycle at the same sagittal level, perpendicular to the long axis of the IVC (a 3D probe may assist in the measurement, thanks to the possibility of imaging long-axis and short-axis IVC simultaneously; Fig. 3A) [15]. It is recommended that an IVC diameter < 2.1 cm, collapsing by more than 50% with inspiration, suggests normal RAP, typically around 3 mmHg (range 0–5 mmHg). Conversely, an IVC diameter greater than 2.1 cm with inspiratory collapse < 50% indicates very high RAP (range 10–20 mmHg) [15]. As a potential limitation, not all patients can tolerate the assessment of IVC, and prolonged training and experience are required to identify and evaluate IVC in most patients [44].

Internal jugular vein (IJV). IJV distension is a proxy for CVP and represents an additional strategy to evaluate systemic venous congestion. Similar to the IVC, IJV distension is associated with a high risk of HF hospitalisation and disease progression [45]. A clinically distended IJV is a powerful marker of poor prognosis in patients with heart failure [45]. However, estimating JVP only by physical exam is often challenging, as the reliability of the evaluation depends on the clinician’s experience, examination time, body size, and intra-thoracic pressure fluctuations [46]. US-based IJV evaluation allows for precise assessment of the diameter of the IJV and its variations during the Valsalva manoeuvre [10] and identifies patients with higher RAP at greater risk [45, 47].

The right IJV lies close to the carotid artery beneath the sternocleidomastoid muscle, allowing easy access for ultrasound examination. For precise evaluation, the patient is studied in a semi-supine position, with the head and neck elevated at approximately 45 degrees, using a high-frequency linear transducer positioned below the jaw angle along the sternocleidomastoid muscle, moving downward toward the angle of Louis [10]. Evaluations performed with the patient in the supine or sitting position should be avoided as they are associated with overdistension and collapse of the IJV, respectively. During the US examination, the diameter of the IJV is measured both at rest and during the Valsalva manoeuvre, either with M-mode or 2D-US (Fig. 3B). The JVD (Jugular Venous Distensibility) ratio is also calculated as the ratio of the IJV diameter during Valsalva to the IJV diameter at rest; this parameter is consistent across different observers [10]. In euvolemic patients with HF, the resting IJV diameter is usually ~ 0.15 cm and increases 5–6 times (i.e., up to 1 cm) during a Valsalva manoeuvre [48, 49]. The maximal diameter achievable during Valsalva remains largely consistent across patients due to the limited compliance of the vessel. Therefore, fluid overload is associated with increased IJV diameter at rest and, in turn, a lower JVD ratio (< 4). [39, 48, 49]. A reduced JVD ratio correlates with more severe symptoms, elevated natriuretic peptides, right ventricular dysfunction, and tricuspid regurgitation [48], but not with LVEF. It also predicts an increased risk of HF-related hospitalisations and death independently of natriuretic peptides [39, 46, 49]. Furthermore, some studies have suggested that the cross-sectional area of the IJV, measured during the Valsalva manoeuvre, might be a more reliable index of RAP [50, 51]. Again, significant variations in the IJV area during Valsalva usually indicate normal CVP and are associated with better prognostic outcomes [50, 51].

One potential limitation of the US evaluation of the IJV is the difficulty in avoiding excess pressure with consequent IJV compression during the examination [52]. This issue may affect the reliability of the measurements and introduce interobserver and intraobserver variability, especially when estimating the volume status and fluid responsiveness in critically ill patients. Therefore, care must be taken to apply the minimum pressure required for the examination [52]. Furthermore, the Valsalva manoeuvre, crucial for effective measurement, can be particularly challenging for acutely unwell patients. For such cases, alternative techniques such as passive leg raising might be considered to estimate CVP, though their impact on the IJV diameter needs further investigation.

**Fig. 4:**
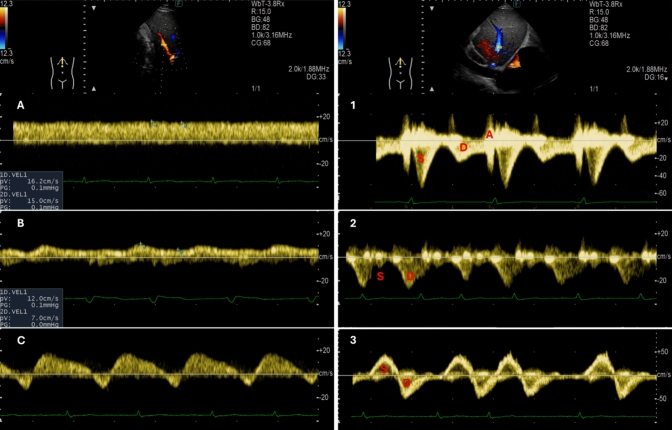
Right. Doppler evaluation of portal vein flow (PVF).** A** Healthy subject; PVF pulsatility fraction = 7%. **B** Outpatient with HF with mildly abnormal PVF (pulsatility fraction = 42%.). **C** Patient hospitalised due to acute HF showing systolic flow reversal (hepatofugal PVF). HF: heart failure. PVF: portal venous flow. Left. Doppler evaluation of hepatic venous flow (HVF). (**1**) Healthy subject with typical triphasic HVF: the S-wave is higher than the D-wave, with a brief A-wave generated by atrial contraction. (**2**) Patient with heart failure and atrial fibrillation, showing a blunted S-wave without the A-wave. (**3**) Patient with severe tricuspid regurgitation and atrial fibrillation, showing a reversed S-wave without the A-wave

Liver US. Doppler evaluation of venous blood flow from the splanchnic district to the liver (i.e., portal venous flow, PVF) and from the liver to the heart (i.e., hepatic venous flow, HVF; Fig. 4) refines the characterisation of systemic congestion and its impact on abdominal organs in HF [16, 53]. Under normal conditions, due to the distensible nature of the hepatic veins, HVF is pulsatile, reflecting the dynamic changes in RAP with each cardiac cycle [16]. The typical waveform observed in the hepatic veins is triphasic, with S- and D-waves directed towards the heart (and away from the liver), indicating systolic and early diastolic right atrial filling; the S-wave is physiologically higher than the D-wave. During late diastole, in the absence of arrhythmias such as atrial fibrillation, the atrial contraction generates a brief A-wave directed towards the liver (and away from the heart). Since venous wall distensibility diminishes as the distance from the right atrium increases, venous flow becomes continuous in more peripheral systemic veins. In particular, the portal vein is at least partially insulated from the right atrium by the resistance of hepatic sinusoids; as a consequence, physiological PVF is continuous or only mildly pulsatile and displays a hepatopetal direction (i.e., it is directed towards the liver) [16, 53]. However, in the presence of worsening venous congestion, the compliance of the IVC reaches a plateau, and the amount of pressure transmitted upstream from the right atrium increases. This results in venous dilation and loss of the physiologic triphasic waveform in the hepatic veins, with a blunted or even reversed S-wave (especially if significant tricuspid regurgitation is present) and the appearance of a positive V-wave indicating right atrial overfilling with subsequent flow recoil towards the liver. Likewise, a shift from continuous to pulsatile PVF may appear, possibly with a brief flow reversal phase (i.e., hepatofugal PVF) during systole in most severe cases [54]. The severity of the alterations in flow dynamics in the portal vein can be quantitatively assessed using parameters like the pulsatility fraction, calculated as the difference between the maximal and minimal PVF velocities, divided by maximal PVF velocity and expressed as a percentage. While no agreed-upon cut-points exist for this parameter, an increased PVF pulsatility fraction has been associated with right ventricular dysfunction and increased CVP [16, 55, 56].

The patient is typically supine or in left lateral decubitus for Doppler evaluation of the hepatic and portal veins. Liver vessels can be identified by positioning a curvilinear or phased array probe in the mid-to-posterior axillary line with the probe marker directed toward the patient’s head and color Doppler imaging with the flow scale adjusted to low-flow velocities (preferably ≤ 20 cm/s) [16]. Measurements should be performed during a breath-hold to mitigate any distortions caused by breathing [16]. Doppler evaluation of the HVF and PVF presents specific challenges. Even in patients with severe tricuspid regurgitation, HVF might not be impaired if the compliance of the right atrium is maintained; this indicates that the evaluation of HVF could potentially underestimate the degree of venous congestion [57]. On the other hand, pulsatile PVF can be observed in healthy, thin individuals and patients with arteriovenous malformations [57]. Also, patients with increased liver stiffness, e.g., liver cirrhosis or metabolic dysfunction-associated steatotic liver disease, may have a non-pulsatile PVF despite severe venous congestion due to a lack of pressure transmission from the right atrium through the liver sinusoid [57].

**Fig. 5:**
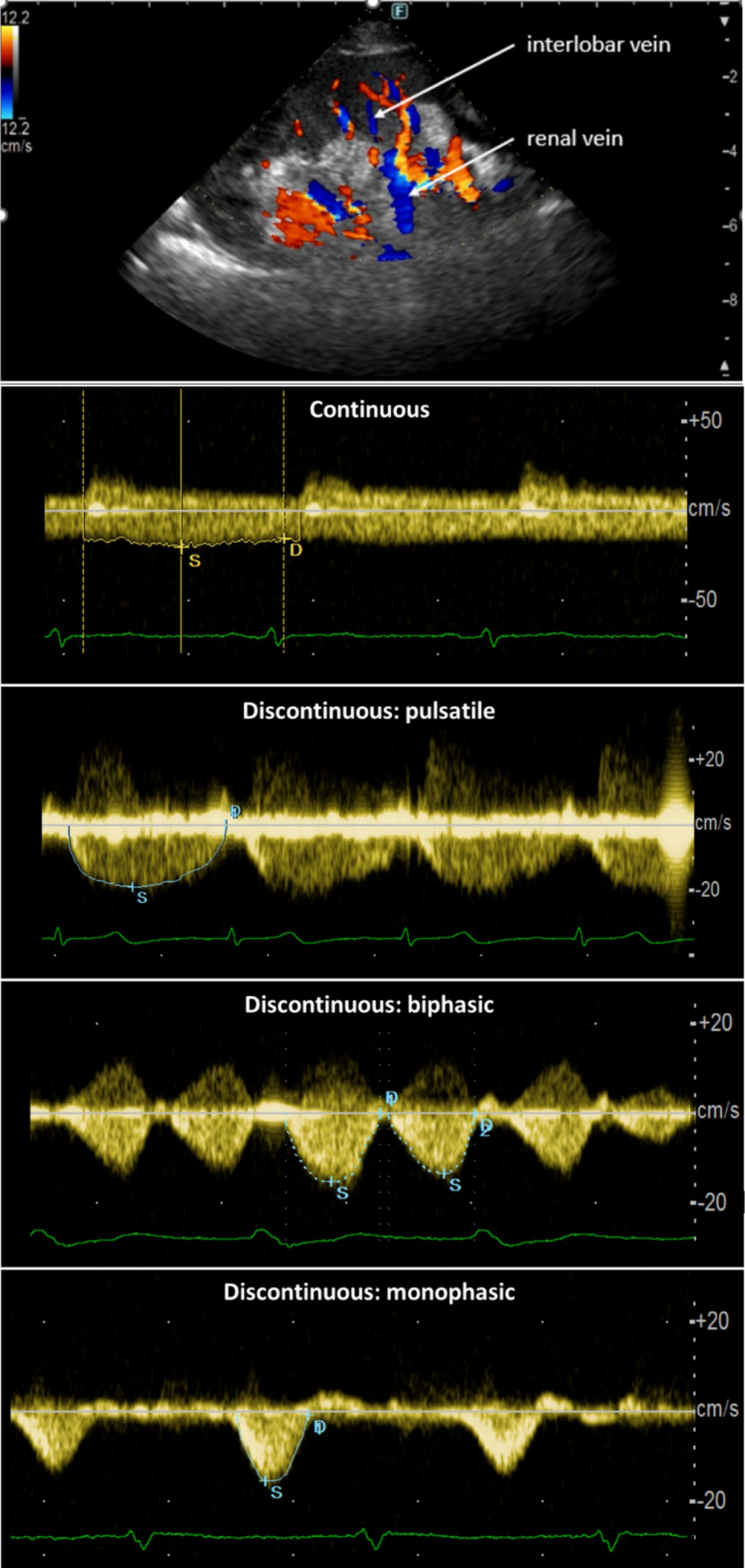
Doppler-derived renal venous flow (RVF) patterns associated with increasing congestion

Renal venous flow (RVF). Doppler-derived evaluation of RVF is emerging as a valuable tool in managing HF, though its clinical usefulness is still under investigation [10]. In euvolemic patients, RVF is continuous, with negligible fluctuations during the cardiac cycle. As CVP increases and intravascular congestion worsens, these fluctuations increase in magnitude, resulting in discontinuous RVF, which can be further categorised qualitatively as pulsatile, biphasic, or even monophasic (Fig. 5). Quantitative metrics such as the venous impedance index (VII) and venous discontinuity index (VDI) further refine the characterisation of renal congestion. The VII is calculated as the difference between the maximum and minimum flow velocities, divided by the maximum flow velocity and expressed as a percentage [3, 10]. Thus, a higher VII indicates increased impedance, with a score of 0 denoting uniform flow and 1 indicating complete flow interruption during the cardiac cycle [3, 10]. Conversely, the VDI is defined as the proportion of the cardiac cycle where flow is absent, again expressed as a percentage [10]. Abnormal RVF patterns correlate with heightened CVP and poorer prognosis in both acute and chronic HF [58, 59]. This is not surprising, as in the presence of elevated CVP, pressure in the interstitium of the encapsulated kidneys is also raised [54], which may lead to further deregulated release of neurohormones, partial collapse of the nephrons, and renal ischaemia, increasing the risk of acute kidney injury [60, 61, 10]. Interestingly, changes in RVF may precede alterations in other echographic indices of congestion, highlighting RVF as a potential early indicator of fluid overload and diuretic response [10].

The patient is placed in the left lateral decubitus position to perform Doppler-derived RVF acquisition and the right kidney is scanned longitudinally during an end-expiratory breath-hold [3], with a convex transducer or phased array aligned at the lowest intercostal view [10]. Color-Doppler imaging (set for peak velocities ≤ 20 cm/s) allows the identification of interlobar veins. The ECG signal is useful for synchronising the RVF signal with the cardiac cycle, ensuring precise timing of measurements [3]. Noteworthy, RVF evaluation may be technically challenging and result in suboptimal recordings if the sonographer is unfamiliar with the technique, albeit we observed a rapid learning curve [3]. Additionally, intrinsic renal diseases could alter intrarenal Doppler venous waveforms, further complicating the interpretation of results [57].

## Conclusions: gap of knowledge and next steps

Identifying and effectively managing fluid overload are critical steps to improve the well-being and prognosis of patients with HF [4]. US is widely available, and several techniques have emerged as reliable, radiation-free, low-cost, non-invasive solutions to evaluate congestion in patients with HF (Table 1). Particularly, serial LUS and IVC evaluations are emerging as valuable tools to tailor diuretic therapy in HF, with encouraging results from preliminary randomised controlled trials [62]. Furthermore, US can reduce the need for radiation-based imaging techniques like chest X-rays and CT scans, lowering radiation exposure, patient transport needs, and hospital costs [35]. However, these techniques are limited by constraints that can hinder their utility in specific situations. Such limitations could be offset by integrating multiple US findings in scoring systems such as the Venous Excess UltraSound (VExUS) scoring system [16, 54]. Indeed, the VExUS score, which incorporates US evaluation of the IVC, HVF, PVF, and RVF, has been proposed to refine the assessment of systemic congestion and enhance clinical decision-making, especially in intensive care settings [16, 63]. Recent findings suggest that specific markers included in the VExUS score—such as intra-renal patterns and PV pulsatility—can improve risk stratification, albeit evidence is still insufficient to support the routine use of the VExUS score to guide decongestion (64). Given that a multi-organ approach can be time-consuming, further research should focus on optimising the combination of ultrasound techniques to develop the most efficient diagnostic algorithm and ultimately improve patient outcomes.

**Table 1:** US techniques used to assess congestion in HF

US technique	How to assess	Evidence	Limitations
Cardiac [10, 12, 17, 18, 24, 65]	• Probe: phased array • Standardised protocols to evaluate cardiac structure and functional anomalies • Can be performed during exercise	• Indirect estimation of pulmonary pressures, LVFP and diastolic function [17, 18]	• Poor acoustic windows (e.g., obese patients, lung disease, *et cetera*) • Pressures are indirectly estimated (limited accuracy compared to RHC) [12]
Lung ultrasound [10, 22, 29, 30]	• Probe: phased array, convex, or linear [14] • Transducer perpendicular or transverse to the ribs • Multiple protocols (8 zones more widely used) • Can be performed during exercise	• Direct relationship with extravascular lung water in HF [23] • Higher sensitivity and specificity than physical exam or chest X-ray in acute dyspnoea [28] • Helps differentiate between HF and other causes of dyspnoea • Correlates with disease severity and risk of readmission or death in HF [39]	• B-lines distribution is influenced by patient position and pleural effusions • B-lines may also be present in non-cardiogenic pulmonary oedema and interstitial lung disease [30] • Obesity is associated with a low number of B-lines
Inferior vena cava	• Probe: phased array or convex • Measurement of IVC diameter in a supine position, subcostal long-axis view, 1–2 cm proximal to the IVC-right atrium junction	• Euvolemia: IVC diameter < 2.1 cm with > 50% inspiratory collapse [15] • IVC diameter changes correlate with RAP changes • Used to monitor diuretic response and predict outcomes	• Weak correlation with RAP in mechanically ventilated patients, increased intra-abdominal pressure, or advanced pulmonary hypertension[40] • Prediction of outcome based on IVC size can be inconsistent
Renal venous flow	• Probe: phased array or convex • Left lateral decubitus position • Qualitative (continuous vs discontinuous) and quantitative (VII and VDI) assessment	• Euvolemia: continuous RVF [3, 10] • Discontinuous RVF suggests high RAP and poor prognosis [58, 59] • Discontinuous RVF and abnormal VII/VDI may precede alterations in estimated CVP • Discontinuous RVF is an early marker of congestion and predicts response to diuretic therapy [10]	• Technically challenging • May be affected by increased abdominal pressure and other renal conditions [57] • Limited large-scale studies on its role in the management of chronic HF
Internal jugular vein	• Probe: linear • Semi-supine position (head and neck elevated at ≈45 degrees), avoiding IJV compression with the probe • Measure IJV diameter at rest and during the Valsalva manoeuvre (M-mode or 2D) to calculate JVD ratio	• Euvolemia: JV diameter < 0.2 cm and JVD ratio > 4 [48, 49] • Increased JV diameter and JVD ratio < 4 correlate with fluid overload and increased RAP [39, 48, 49] • Low JVD ratio predicts worse HF outcomes	• Inability to perform Valsalva manoeuvre [10]
Liver [16, 57]	• Probe: phased array or convex • Supine or left lateral decubitus position • Evaluation of PVF and HVF	• Euvolemia: PVF is continuous or mildly pulsatile; HV with S-wave higher than D-wave [16, 53] • With increasing RAP, PVF becomes pulsatile; in the hepatic veins, S-wave is blunted or even reversed [54]	• Technically challenging • HVF is unaltered if RA compliance is maintained, even in congested patients • Pulsatile PVF in healthy, thin individuals and patients with arteriovenous malformations • Increased liver stiffness may limit the reliability of PVF [57]

*HF* heart failure, *HVF* hepatic venous flow, *IVC* inferior vena cava, *IJV* internal jugular vein, *JVD* jugular venous distention, *LUS* lung ultrasound, *LVFP* left ventricular filling pressure, *PVF* portal venous flow, *PCWP* pulmonary capillary wedge pressure, *RA* right atrium, *RAP* right atrial pressure, *RHC* right heart catheterisation, *RVF* renal venous flow, *US* ultrasound, *VDI* venous distensibility index, *VII* venous impedance index
